# Correlations and validations of dual-bolus and dual-sequence quantification of first-pass myocardial perfusion CMR in humans and canines

**DOI:** 10.1186/1532-429X-18-S1-Q17

**Published:** 2016-01-27

**Authors:** Li-Yueh Hsu, Peter Kellman, Peter Gatehouse, Hannah M Conn, Mitchel Benovoy, Matthew Jacobs, Andrew E Arai

**Affiliations:** 1 grid.94365.3d0000000122975165National Heart Lung and Blood Institute, National Institutes of Health, Bethesda, MD USA; 2 grid.439338.6Royal Brompton Hospital, London, United Kingdom; 3 grid.183158.60000000404353292Polytechnique Montreal, Montreal, QC Canada; 4 grid.39936.360000000121746686Catholic University of America, Washington, DC USA

## Background

Dual-bolus and dual-sequence techniques have been proposed to maintain the linearity of arterial input function (AIF) in LV during first-pass CMR perfusion imaging. This study compared myocardial blood flow (MBF) estimates using both techniques in humans and in a canine model.

## Methods

CMR perfusion imaging was performed in six canines and thirty patients at 1.5T using dual-bolus (0.005 and 0.05 mmol/kg Gd-DTPA) and dual-sequence techniques with 1RR, 90° composite pulse, 50° SSFP readout, saturation recovery 90 ms, TR 2.4 ms, TE 1.2 ms, matrix size 128 × 80. A low TE 0.6 ms, low-resolution 64 × 48 FLASH image series was also acquired. The AIF was measured from the low-dose high-resolution series (DB), the high-dose low-resolution series (DS), and the high-dose high-resolution conventional single-bolus series (SB). Myocardial time intensity curves were analyzed on a mid-slice based on 6 transmural sectors and quantified by model-constrained deconvolution.

## Results

In canine experiments, the Pearson's correlation between microsphere MBF and DB (r = 0.89, figure-a) and DS (r = 0.89, figure-b) estimates were excellent with small bias in Bland-Altman analysis (bias -0.19 and -0.73 ml/g/min). There was an excellent correlation and reasonable bias between DB and DS estimates of MBF in canines (r = 0.97, figure-c) and patients (r = 0.98, figure-d). However, SB overestimated MBF (bias +2.50 ml/g/min, p < 0.001) despite a good correlation with microspheres (r = 0.88). In human studies, SB also overestimated MBF versus either DB or DS estimates (bias +1.47 and +1.38 ml/g/min, p < 0.001).

## Conclusions

The MBF estimates by DB and DS are suitable for CMR perfusion quantification. However, SB experiments have large errors in MBF quantification with the doses and parameters studied.

**Figure 1:**
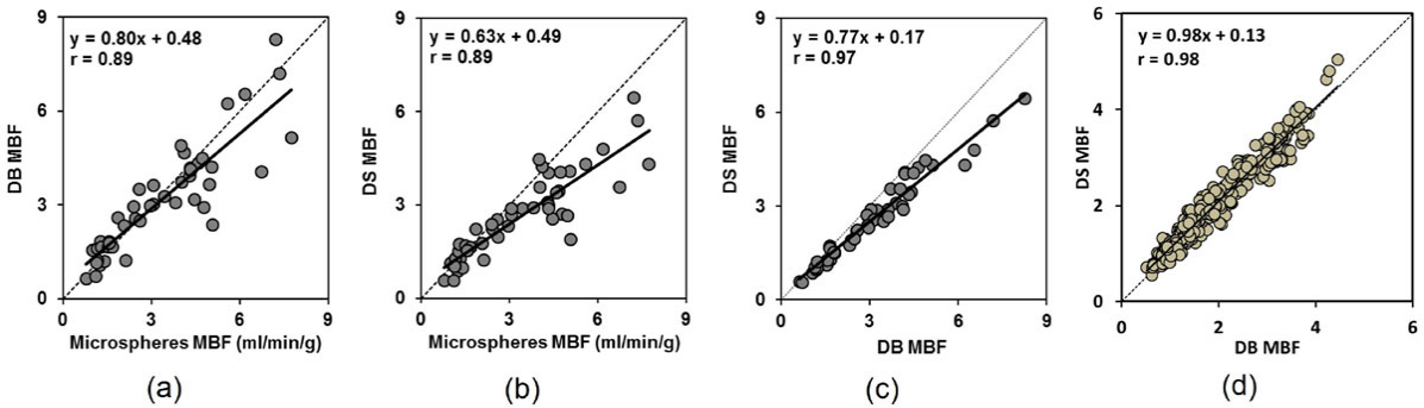
Figure 1

